# Pre-pregnancy obesity, not gestational weight gain, is a high-risk factor for pre-eclampsia: a retrospective study in Bengbu, China

**DOI:** 10.7717/peerj.21216

**Published:** 2026-05-14

**Authors:** Jie Gao, Yu Wang

**Affiliations:** 1 Department of Pathology, The Third People’s Hospital of Bengbu, Bengbu Medical University, Bengbu, Anhui, China; 2 Department of Pathology, Women’s Hospital, School of Medicine, Zhejiang University, Hangzhou, China; 3 Department of Obstetrics and Gynecology, The Third People’s Hospital of Bengbu, Bengbu Medical University, Bengbu, Anhui, China; 4 Department of Obstetrics and Gynecology, Women’s Hospital, School of Medicine, Zhejiang University, Hangzhou, China

**Keywords:** Body mass index, Gestational weight gain, Maternal obesity, Pre-Eclampsia, Risk

## Abstract

**Objective:**

To examine the prevalence of obesity among full-term pregnant women and investigate its etiological association with pre-eclampsia through a retrospective study.

**Methods:**

Data from 2,153 singleton full-term pregnant women were categorized into four groups based on their pre-pregnancy body mass index (PBMI): Underweight, Healthy weight, Overweight, and Obesity. The incidence rate and relative risk of pre-eclampsia were calculated in each group. Gestational weight gain (GWG) was compared across groups, and women were classified as having no excess or excess GWG according to the 2009 Institute of Medicine guidelines. The proportion and relative risk of excess GWG were calculated for each group, as were the incidence rate and relative risk of pre-eclampsia in relation to excess GWG. The associations between pre-eclampsia severity and each of PBMI and GWG were assessed.

**Results:**

The analysis revealed differential pre-eclampsia incidence across PBMI classifications: Underweight (2.78%), Healthy weight (4.66%), Overweight (13.97%), and Obesity (25.35%). Compared with Healthy weight, the relative risk (95% confidence intervals) of pre-eclampsia in Underweight, Overweight, and Obesity were 0.596 (0.262, 1.354), 2.996 (2.128, 4.219), and 5.436 (3.432, 8.621), respectively. Mean GWG was 15.22 ± 4.77 kg. GWG was significantly higher among Underweight and Healthy weight women than among Overweight and Obesity women (all *P* < 0.001). Excess GWG exhibited an ascending pattern across BMI categories: Underweight (27.31%), Healthy weight (41.31%), Overweight (63.01%), and Obesity (76.06%). Among the 2,153 pregnant women, PBMI was positively correlated with PE severity, whereas GWG showed no significant association. The estimated regression coefficients (95% confidence intervals) were 0.225 (0.179, 0.271) and −0.030 (−0.065, 0.005), respectively.

**Conclusions:**

Pre-pregnancy obesity is a high-risk factor for pre-eclampsia, whereas an independent association with excessive gestational weight gain was not demonstrated in this cohort. Controlling pre-pregnancy obesity remains a key focus in obstetrics.

## Introduction

Pre-eclampsia (PE) is one of the most serious pregnancy-related complications for both the mother and fetus. Obesity is significantly associated with numerous obstetric complications, such as PE, gestational diabetes mellitus, increased cesarean section rates, macrosomia, and small gestational age (Xiang et al., 2024; Nagler et al., 2024; Addicott et al., 2024; Girchenko et al., 2024; Wahabi et al., 2024; Sun et al., 2023; Witteveen et al., 2022; Heslehurst et al., 2022; Frey et al., 2022; Liu et al., 2022; Tanner, Brock And & Chauhan, 2022; Li et al., 2022; 13, 2021; Otero-Naveiro et al., 2021; Lauth et al., 2021; Vince et al., 2021; Cosson et al., 2020; Bicocca et al., 2020; Brown, Kapurubandara & McGee, 2020; Pratt, Howat & Hui, 2020; Siddiqui et al., 2020; He et al., 2020; Foroozanfard et al., 2020; Meghelli et al., 2020; Fallatah et al., 2019; Alves et al., 2019; Melchor et al., 2019; Marshall et al., 2019; Bender, Hirshberg & Levine, 2019). The weight of a pregnant woman consists of two components: pre-pregnancy baseline weight and gestational weight gain (GWG). Previous studies have primarily identified obesity as a significant risk factor for PE. However, it remains unclear whether pre-pregnancy obesity or excessive GWG is strongly associated with the incidence of PE. This study aimed to address this gap by examining the weight distribution of full-term pregnant women in our hospital, both before pregnancy and prior to delivery, over a 1-year period. We analyzed the relationship between pre-pregnancy obesity, GWG, and PE to determine whether women who were obese before pregnancy experienced greater GWG and whether PE was more closely associated with pre-pregnancy obesity or excessive GWG during pregnancy.

## Materials & Methods

The Third People’s Hospital of Bengbu granted ethical approval for this retrospective study (Ethical Application Ref: 2023-K57). All patients provided written informed consent for the collection and publication of their clinical data. The inclusion criteria were singleton, full-term pregnant women who gave birth at our hospital between July 1, 2022, and June 30, 2023. The exclusion criteria included pregnant women unable to provide accurate weight and height data, as well as those with elevated blood pressure prior to 20 weeks of gestation, to eliminate the influence of pre-existing hypertension on PE. PE and severe PE were diagnosed according to the criteria outlined in the 10th Edition of the Chinese Textbook of Obstetrics and Gynecology (Kong, Ma & Duan, 2024), which aligns with the 2020 American College of Obstetricians and Gynecologists Practice Bulletin (Anonymous, 2020).

A total of 2,153 pregnant women were included in the study after applying the inclusion and exclusion criteria. Pre-pregnancy weight and height were self-reported. Self-reported pre-gestational weight aligned well with early pregnancy measurements recorded in the maternity health care booklet, and self-reported height was consistent with obstetricians’ assessments. Participants were categorized into four groups according to their pre-pregnancy body mass index (PBMI): Underweight (BMI < 18.5 kg/m^2^), Healthy weight (18.5 ≤ BMI < 25 kg/m^2^), Overweight (25 ≤ BMI < 30 kg/m^2^), and Obesity (BMI ≥ 30 kg/m^2^). Incidence rates (IRs) and relative risks (RRs) with 95% confidence intervals (CIs) for PE were calculated for each group.

Differences in GWG among the four groups were compared. Based on the 2009 Pregnancy Weight Guidelines from the Institute of Medicine (IOM) (Rasmussen, Yaktine & editors, 2009), pregnant women were classified as having “No Excess GWG” or “Excess GWG”. Within each group, the IR for “Excess GWG” was calculated, and the IRs for PE in the “No Excess GWG” and “Excess GWG” groups were compared. Furthermore, the RR (95% CI) for PE in “Excess GWG” compared with “No Excess GWG” was calculated. Associations between the severity of PE (severe PE, mild PE, and no gestational hypertension) and PBMI, as well as GWG, were analyzed.

Statistical analyses were performed using IBM SPSS Statistics software (version 27; IBM Corp., Armonk, NY, USA). For normally distributed data, the mean ± standard deviation was used. For non-normally distributed data, medians (interquartile ranges) were used. Because the measurement data from each group did not satisfy the assumptions of normality and homogeneity of variance, the Kruskal–Wallis test (nonparametric one-way analysis of variance (ANOVA) test (k samples) was used to compare medians. *Post-hoc* pairwise comparisons between groups were conducted using Bonferroni correction to adjust for multiple comparisons. IRs were compared using Pearson’s chi-square test. The RRs (95% CIs) were calculated using the cross-tabulation risk estimate. Ordinal logistic regression was employed to assess the associations between PBMI, GWG, maternal age, parity, and gravidity and the severity of PE (severe PE, mild PE, and no gestational hypertension).

## Results

### Pre-pregnancy overweight and obesity are significant risk factors for PE

Among the 2,153 pregnant women surveyed, 216 (10.03%) were Underweight, 1,501 (69.72%) were Healthy weight, 365 (16.95%) were Overweight, and 71 (3.30%) were Obesity. Using the Kruskal–Wallis one-way ANOVA test (k samples), we found no statistically significant differences in age, gravidity, or parity among the four groups. Pearson’s chi-square test revealed a significant difference in the IR of PE among the four groups (the IRs of PE in the four groups were 2.78%, 4.66%, 13.97%, and 25.35%, respectively; *P* < 0.001), and pairwise comparisons between the groups identified significant differences for most contrasts (*P* < 0.05). Using cross-tabulation risk estimates, the RRs (95% CIs) of PE for each group relative to Healthy weight group were calculated. Compared with Healthy weight, the RRs (95% CIs) of PE for Underweight, Overweight, and Obesity were 0.596 (0.262, 1.354), 2.996 (2.128, 4.219), and 5.436 (3.432, 8.612), respectively. The general characteristics of the four groups, along with the IRs and RRs (95% CIs) of PE, are presented in Table 1.

**Table 1:** General conditions of the four groups of pregnant women and the incidence rates (IRs) and relative risks with 95% confidence intervals (RRs, 95% CIs) of pre-eclampsia (PE).

Group	*N*	Age (year)	Gravidity	Parity	PE (IR)	RR (95% CI)
Underweight	216	28 (25, 31)	2 (1, 3)	0 (0, 1)	6 (2.78%)	0.596 (0.262, 1.354)
Healthy weight	1,501	29 (26, 31)	2 (1, 3)	0 (0, 1)	70 (4.66%)	1
Overweight	365	29 (26, 31)	2 (1, 3)	0 (0, 1)	51 (13.97%)	2.996 (2.128, 4.219)
Obesity	71	28 (26, 31)	2 (1, 3)	0 (0, 1)	18 (25.35%)	5.436 (3.432, 8.621)
P		0.055	0.410	0.746	<0.001	

**Notes.**

Following the Kruskal–Wallis one-way ANOVA test (k samples), there were no statistically significant differences in age, gravidity, or parity among the four groups. Pearson’s chi-square test revealed a statistically significant difference in the IR of PE among the four groups (*P* < 0.001), and pairwise comparisons between the groups also showed statistically significant differences (all *P* < 0.05). Using cross-tabulation risk estimates, the RR (95% CI) of PE in Overweight or Obesity pregnant women was found to be statistically significant.

### Statistical distribution of GWG among full-term pregnant women

**Figure 1:**
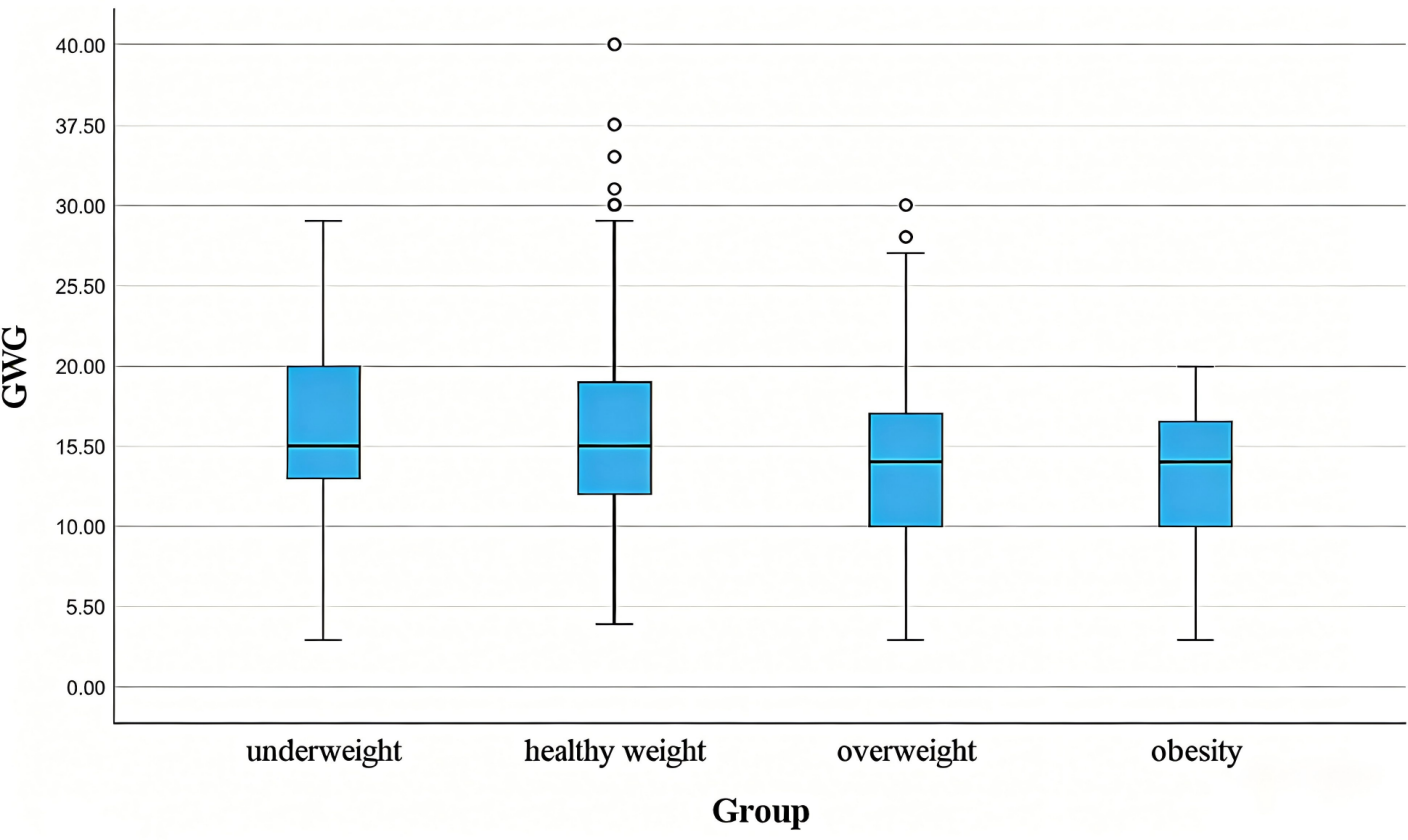
The gestational weight gain (GWG) in the four groups. Underweight and healthy weight pregnant women exhibited more significant GWG than their overweight and obese counterparts.

Among the 2,153 singleton full-term pregnancies, mean GWG was 15.22 ± 4.77 kg. The GWG in the four groups did not exhibit homogeneity of variance. The Kruskal–Wallis one-way ANOVA test (k samples) revealed a statistically significant difference in GWG among the four groups (*P* < 0.001). Pairwise comparisons using the Bonferroni correction revealed no statistically significant difference in GWG between Underweight and Healthy weight pregnant women (15.86 ± 4.38 *vs.* 15.55 ± 4.65, adjusted *P* = 0.999) and no significant difference between Overweight and Obesity pregnant women (13.95 ± 5.08 *vs.* 12.66 ± 4.75, adjusted *P* = 0.901). All other pairwise comparisons between groups were statistically significant (all adjusted *P*-values < 0.001). Underweight and Healthy weight pregnant women exhibited greater weight gain than their Overweight and Obesity counterparts. The GWG across the four groups is shown in Fig. 1.

Currently, authoritative and standardized guidelines for GWG are lacking in China. In accordance with American IOM recommendations, pregnant women were categorized into two groups: No Excess GWG and Excess GWG. Pearson’s chi-square test showed a statistically significant difference in the rate of Excess GWG among the four groups (*P* < 0.001). Furthermore, pairwise comparisons between the groups revealed statistically significant differences (all *P* values < 0.05). The IRs of Excess GWG in the four groups were 27.31%, 41.31%, 63.01%, and 76.06%, respectively. The proportion was lowest in the Underweight group and markedly higher in Overweight and Obesity groups than in Healthy weight group. Using cross-tabulation risk estimates, when compared with Healthy weight pregnant women, the RRs (95% CIs) of Excess GWG for Underweight, Overweight, and Obesity pregnant women were 0.661 (0.528, 0.829), 1.526 (1.382, 1.684), and 1.841 (1.595, 2.126), respectively. The distribution of Excess GWG across the different groups, along with the RR (95% CI) for Excess GWG, is summarized in Table 2.

**Table 2:** The incidence rates (IRs) of excessive weight gain (Excess GWG) and relative risks with 95% confidence intervals (RRs, 95% CIs) in each group of pregnant women.

Group	*N*	GWG (Kg)	Excess GWG		IR	RR (95% CI)
			No	Yes		
Underweight	216	15.86 ± 4.38	157	59	27.31%	0.661 (0.528, 0.829)
Healthy weight	1,501	15.55 ± 4.65	881	620	41.31%	1
Overweight	365	13.95 ± 5.08	135	230	63.01%	1.526 (1.382, 1.684)
Obesity	71	12.66 ± 4.75	17	54	76.06%	1.841 (1.595, 2.126)
P		<0.001			<0.001	

**Notes.**

Pearson’s chi-square test showed a statistically significant difference in the rate of Excess GWG among the four groups (*P* < 0.001). Furthermore, pairwise comparisons between the groups revealed statistically significant differences (all *P* < 0.05). Using cross-tabulation risk estimates, the RR (95% CI) values for excess GWG in each group of pregnant women were statistically significant.

### Etiological investigation into the relationship between excessive GWG and PE

Pearson’s chi-square test and cross-tabulation risk estimates did not show a significant difference in the IRs and RRs for PE between women with Excess GWG and No Excess GWG in each PBMI group. Based on this observation, we believe that in studies with relatively small sample sizes within subgroups, it is difficult to conclusively determine whether excessive GWG is a risk factor for PE. However, among the 2,153 full-term pregnant women studied, the IRs of PE in the Excess GWG and No Excess GWG groups were 8.72% and 5.13%, respectively (*P* = 0.001). Compared with the No Excess GWG group, the RR (95% CI) of PE in the Excess GWG group was 1.702 (1.237, 2.340). The IR and RR (95% CI) for PE among pregnant women with Excess GWG across different groups are summarized in Table 3.

**Table 3:** The incidence rate (IR) and relative risk with 95% confidence interval (RR, 95% CI) of pre-eclampsia (PE) in pregnant women with excessive weight gain (Excess GWG).

Group	Category	Excess GWG	No Excess GWG	*P*	RR (95% CI)
Underweight	PE (cases)	3	3		2.661 (0.552, 12.817)
	not PE (cases)	56	154		
	IR	5.08%	1.91%	0.349	
Healthy weight	PE (cases)	33	37		1.267 (0.802, 2.003)
	not PE (cases)	587	844		
	IR	5.32%	4.20%	0.310	
Overweight	PE (cases)	34	17		1.174 (0.683, 2.019)
	not PE (cases)	196	118		
	IR	14.78%	12.59%	0.560	
Obesity	PE (cases)	14	40		1.031 (0.771, 1.378)
	not PE (cases)	4	13		
	IR	77.78%	75.47%	0.999	
Term pregnant women^a^	PE (cases)	84	61		1.702 (1.237, 2.340)
	not PE (cases)	879	1,129		
	IR	8.72%	5.13%	0.001	

**Notes.**

Pearson’s chi-square test and cross-tabulation risk estimate did not reveal a statistically significant difference in the IR and RR (95% CI) of PE between pregnant women with excess GWG and those without excess GWG in each group. However, within the entire study cohort, the IR and RR values for PE in pregnant women with excess GWG increased significantly.

### Associations between the severity of PE and PBMI, as well as GWG

To correct for confounding factors such as maternal age, parity, and gravidity, ordinal logistic regression was employed to assess the associations between PBMI, GWG, and the severity of PE in each group. The severity of PE was classified as ordinal data, where severe PE was assigned a value of 2, mild PE was assigned a value of 1, and normotension (no gestational hypertension) was assigned a value of 0. The severity of PE was treated as the dependent variable; gravidity and parity were treated as factors; and age, PBMI and GWG were treated as covariates. After testing for parallel lines, the data in each group satisfied the assumptions of ordinal logistic regression. In Underweight group, PBMI was negatively correlated with the severity of PE, whereas GWG was positively correlated with PE severity. The estimated regression coefficients with 95% confidence intervals (β, 95% CI) were −0.987 (−1.801, −0.173) and 0.774 (0.259, 1.289), respectively. In Healthy weight group, a positive correlation was observed between PBMI and PE severity; however, there was no statistically significant association between GWG and PE severity. The corresponding β (95% CI) were 0.243 (0.104, 0.381) and −0.031 (−0.101, 0.040), respectively. For Overweight group, PBMI was positively correlated with PE severity, while an inverse correlation was found between GWG and PE severity. The β (95% CI) were 0.437 (0.219, 0.655) and −0.138 (−0.230, −0.046), respectively. In Obesity group, neither PBMI nor GWG showed a statistically significant association with the severity of PE, with the β (95% CI) being 0.057 (−0.345, 0.459) and 0.041 (−0.147, 0.228), respectively. Ultimately, in the total study population of 2,153 pregnant women, PBMI was positively correlated with the severity of PE, whereas no statistically significant association was found between GWG and PE severity. The β (95% CI) were 0.225 (0.179, 0.271) and −0.030 (−0.065, 0.005), respectively. The estimated regression coefficients of PBMI and GWG associated with PE severity in each pregnant woman group are summarized in Table 4.

**Table 4:** The estimated regression coefficients with 95% confidence intervals (β, 95% CI) for pre-eclampsia severity associated with pre-pregnancy body mass index (PBMI) and gestational weight gain (GWG) in each group of pregnant women.

Group	*N*	P^1^		β(95% CI)	P^2^
Underweight	216	0.999	PBMI-PE	−0.987 (−1.801, −0.173)	0.018
			GWG-PE	0.774 (0.259, 1.289)	0.003
Healthy weight	1,501	0.990	PBMI-PE	0.243 (0.104, 0.381)	0.001
			GWG-PE	−0.031 (−0.101, 0.040)	0.389
Overweight	365	0.999	PBMI-PE	0.437 (0.219, 0.655)	0.001
			GWG-PE	−0.138 (−0.230, −0.046)	0.003
Obesity	71	0.916	PBMI-PE	0.057 (−0.345, 0.459)	0.781
			GWG-PE	0.041 (−0.147, 0.228)	0.672
Term pregnant women	2,153	0.999	PBMI-PE	0.225 (0.179, 0.271)	0.001
			GWG-PE	−0.030 (−0.065, 0.005)	0.093

**Notes.**

To correct for confounding factors such as maternal age, parity, and gravidity, ordinal logistic regression was employed to assess the associations between PBMI, GWG, and the severity of pre-eclampsia in each group.

P^1^ The significance of the Test of Parallel Lines. After testing for parallel lines, the data in each group met the assumptions of ordinal logistic regression.

P^2^ The significance of estimated regression coefficients. In the larger subgroups of the Healthy weight and Overweight, as well as in the overall study population of 2,153 pregnant women, PBMI was positively correlated with the severity of pre-eclampsia, whereas GWG was not statistically significantly associated with pre-eclampsia severity.

## Discussion

We conducted a survey to examine the prevalence and distribution of obesity among full-term pregnant women and investigate its etiological association with PE. Our findings further corroborate the established perspective that obesity is a significant risk factor for PE, which is consistent with extensive clinical evidence. Obesity remains a major risk factor for numerous obstetric complications, including gestational diabetes, macrosomia, pre-term birth, and a higher incidence of cesarean section. These perspectives have been incorporated into Chinese obstetrics and gynecology textbooks. However, the absence of dedicated guidelines addressing obesity in pregnant women suggests a lack of adequate attention among obstetricians in China.

China is a developing country characterized by unbalanced economic and social development. Typically, people in China perceive the prevalence of obesity among women in economically advanced coastal cities to be lower than that in rural areas of the central and western regions. In Bengbu City, a medium-sized city in central China, pregnant women generally have relatively low educational levels, low incomes, and experience little work- or life-related pressure. Moreover, pregnant women in this area lack awareness of diet control and exercise. These factors appear to be associated with female obesity, and such phenomena are prevalent in the central and western regions. Although the differences in obesity rates between Bengbu and Shenzhen (Liu et al., 2022) may not appear striking at first glance, clinical experience in obstetric practice has revealed a notable increase in the number of obese pregnant women in Bengbu, which has negatively affected the vaginal delivery rate and significantly increased the complexity of cesarean section procedures for surgeons. The distribution of obesity among pregnant women in Bengbu may reflect a useful indicator of obesity patterns among pregnant women in certain regions, but confirmation through multi-center studies is needed. According to recent data (Frey et al., 2022; Benham et al., 2021), the prevalence of obesity among pregnant women is notably higher in North America than in China, potentially owing to social, racial, and lifestyle-related factors. Our findings highlight the need to implement preventive measures and make consistent efforts to mitigate the increasing trend of obesity among women in China.

Obesity, the most prevalent metabolic disorder, is attracting increasing attention. Obstetricians typically regard obesity as a high-risk factor for PE. Excessive fat accumulation can directly result in abnormal expression and secretion of adipokines, particularly inflammatory adipokines, which cause metabolic alterations and inflammation within the body. This, in turn, leads to changes in the maternal and placental microcirculation, vascular endothelial dysfunction, systemic small-vessel spasm and remodeling, and ultimately the development of PE (Kivelä et al., 2021; Tabacu et al., 2022; Stupak et al., 2021; Vonck et al., 2019). Therefore, adipokines are considered to play a mediating role between obesity and PE (Faulkner et al., 2024; Moronge et al., 2024; Rumer et al., 2024; Ozmen et al., 2023).

Reasonable control of GWG is essential (Lipworth, 2021; Benham et al., 2021; Mustafa et al., 2022). Under the guidance of obstetricians, overweight and obese pregnant women are informed of the risks associated with obesity and are actively guided to manage their weight gain. Although no universally accepted guidelines for weight gain during pregnancy exist in China, many obstetricians recommend a total increase of ≤10 kg during pregnancy. Some obstetricians consider zero weight gain acceptable for women who are severely obese before pregnancy. The IOM recommendations used in this study impose stricter GWG limits on overweight and obese pregnant women, resulting in a higher proportion of women falling within the Excess GWG category. However, in practice, the median GWG for overweight and obese pregnant women remains lower than that for underweight and healthy weight pregnant women. These data suggest that obesity-associated PE is predominantly related to PBMI. A potential association between GWG and PE may exist only among pregnant women with insufficient pre-pregnancy weight. Our findings indicate that the elevated risk of PE in pregnant women who are overweight and obese is primarily attributable to pre-pregnancy overweight status and obesity rather than excessive GWG. Consequently, we cannot conclude that excessive GWG is an independent risk factor for PE after accounting for pre-pregnancy BMI. This observation suggests that obstetricians in this region have effectively educated pregnant women on the management of GWG.

Most severe PE cases result in either spontaneous pre-term birth or iatrogenic pre-term deliveries. Because the present study focused solely on full-term pregnancies, it likely underestimates the etiological relationship between obesity and severe PE, as many severe PE cases were not captured. Obesity in pregnant women warrants recognition as an independent factor and should be incorporated into Chinese obstetrics and gynecology textbooks. This initiative should begin with obstetricians’ education and emphasize the detrimental effects of obesity on women’s reproductive health. Obstetricians have effectively educated pregnant women, and this effort must be sustained. Chinese medical and health professionals should continue to focus on educational initiatives aimed at reducing the prevalence of overweight and obesity in pregnant women. Promoting pre-pregnancy weight management across society is a valuable and worthwhile direction for ongoing obstetric efforts (Addicott et al., 2024; Vince et al., 2021; Cosson et al., 2020; Pratt, Howat & Hui, 2020; Alves et al., 2019; Sharma, Blumenthal & Poppas, 2021; Ogunwole, Zera & Stanford, 2021).

Despite the valuable insights generated, this study has certain limitations. First, its retrospective design relied on existing hospital records, which may have been subject to measurement errors and incomplete data. The possibility of residual measurement errors remains, particularly for self-reported or non-standardized measurements. Second, because our sample was derived from a single institution and was limited to full-term singleton pregnancies, the findings may not be generalizable to pre-term pregnancies, populations in other regions, or clinical settings. Third, although our statistical methods (including nonparametric tests and Bonferroni-adjusted *post-hoc* comparisons) were chosen because of violations of normality and variance homogeneity, these approaches may have reduced the statistical power relative to parametric tests. Finally, potential confounding variables, such as gestational diabetes, socioeconomic status, nutritional intake, and other comorbidities, were not fully controlled, which could have influenced both GWG and the development of PE. These limitations should be considered when interpreting the etiological relationships observed in this study.

## Conclusions

Currently, pre-pregnancy obesity is a high-risk factor for PE; however, an independent effect of excessive GWG was not demonstrated in this study. Sustained and targeted interventions are essential to educate women of childbearing age on the critical importance of achieving and maintaining a healthy weight before conception.

##  Supplemental Information

10.7717/peerj.21216/supp-1 Supplemental Information 1 Original data The weight indicators of 2,153 pregnant women and their association with preeclampsia.

## Additional Information and Declarations

### Data Availability

The following information was supplied regarding data availability: The raw data are available in the Supplemental File.

## References

[ref-1] Addicott K, Nudelman M, Putty K, Prasher P, Preston D, Yoost JL, De Fruscio A, Bartlett D, Cavender C, Carter M, Datz H, Rodriquez K, Werthammer J (2024). Adverse perinatal outcomes associated with increasing maternal obesity. American Journal of Perinatology.

[ref-2] Alves P, Malheiro MF, Gomes JC, Ferraz T, Montenegro N (2019). Risks of maternal obesity in pregnancy: a case-control study in a Portuguese obstetrical population. Revista Brasileira de Ginecologia e Obstetrícia.

[ref-3] (2020). Gestational hypertension and preeclampsia: ACOG practice bulletin, number 222. Obstetrics and Gynecology.

[ref-4] Bender W, Hirshberg A, Levine LD (2019). Interpregnancy body mass index changes: distribution and impact on adverse pregnancy outcomes in the subsequent pregnancy. American Journal of Perinatology.

[ref-5] Benham JL, Booth JE, Donovan LE, Leung AA, Sigal RJ, Rabi DM (2021). Prevalence of and risk factors for excess weight gain in pregnancy: a cross-sectional study using survey data. CMAJ Open.

[ref-6] Bicocca MJ, Mendez-Figueroa H, Chauhan SP, Sibai BM (2020). Maternal obesity and the risk of early-onset and late-onset hypertensive disorders of pregnancy. Obstetrics and Gynecology.

[ref-7] Brown J, Kapurubandara S, McGee TM (2020). Confounding effect of ethnic diversity on booking-in body mass index and prevalence of gestational diabetes and hypertensive disorders in pregnant women in western Sydney 1997–2016. Australian and New Zealand Journal of Obstetrics and Gynaecology.

[ref-8] Cosson E, Vicaut E, Sandre-Banon D, Gary F, Pharisien I, Portal JJ, Baudry C, Cussac-Pillegand C, Costeniuc D, Valensi P, Carbillon L (2020). Performance of a selective screening strategy for diagnosis of hyperglycaemia in pregnancy as defined by IADPSG/WHO criteria. Diabete Et Metabolisme.

[ref-9] Fallatah AM, Babatin HM, Nassibi KM, Banweer MK, Fayoumi MN, Oraif AM (2019). Maternal and neonatal outcomes among obese pregnant women in King Abdulaziz University Hospital: a retrospective single-center medical record review. Medical Archives.

[ref-10] Faulkner JL, Takano M, Ogbi S, Tong W, Nakata M, Moronge D, Cindrova-Davies T, Giussani DA (2024). Mid-late gestation leptin infusion induces placental mitochondrial and endoplasmic reticulum unfolded protein responses in a mouse model of preeclampsia. Placenta.

[ref-11] Foroozanfard F, Asemi Z, Bazarganipour F, Taghavi SA, Allan H, Aramesh S (2020). Comparing pregnancy, childbirth, and neonatal outcomes in women with different phenotypes of polycystic ovary syndrome and healthy women: a prospective cohort study. Gynecological Endocrinology.

[ref-12] Frey HA, Ashmead R, Farmer A, Kim YH, Shellhaas C, Oza-Frank R, Jackson RD, Costantine MM, Lynch CD (2022). Association of prepregnancy body mass index with risk of severe maternal morbidity and mortality among medicaid beneficiaries. JAMA Network Open.

[ref-13] Girchenko P, Lahti-Pulkkinen M, Hämäläinen E, Laivuori H, Villa PM, Kajantie E, Räikkönen K (2024). Associations of polymetabolic risk of high maternal pre-pregnancy body mass index with pregnancy complications, birth outcomes, and early childhood neurodevelopment: findings from two pregnancy cohorts. BMC Pregnancy and Childbirth.

[ref-14] He Y, Tian J, Blizzard L, Oddy WH, Dwyer T, Venn AJ (2020). Associations of childhood adiposity and changes in adiposity status from childhood to adulthood with pregnancy hypertension. Pregnancy Hypertension.

[ref-15] Heslehurst N, Ngongalah L, Bigirumurame T, Nguyen G, Odeniyi A, Flynn A, Smith V, Crowe L, Skidmore B, Gaudet L, Simon A, Hayes L (2022). Association between maternal adiposity measures and adverse maternal outcomes of pregnancy: systematic review and meta-analysis. Obesity Reviews.

[ref-16] Kivelä J, Sormunen-Harju H, Girchenko PV, Huvinen E, Stach-Lempinen B, Kajantie E, Villa PM, Reynolds RM, Hämäläinen EK, Lahti-Pulkkinen M, Murtoniemi KK, Laivuori H, Eriksson JG, Räikkönen K, Koivusalo SB (2021). Longitudinal metabolic profiling of maternal obesity, gestational diabetes, and hypertensive pregnancy disorders. Journal of Clinical Endocrinology and Metabolism.

[ref-17] Kong B, Ma D, Duan T (2024). Obstetrics and gynecology.

[ref-18] Lauth C, Huet J, Dolley P, Thibon P, Dreyfus M (2021). Maternal obesity in prolonged pregnancy: labor, mode of delivery, maternal and fetal outcomes. Journal of Gynecology Obstetrics and Human Reproduction.

[ref-19] Li MF, Ke JF, Ma L, Wang JW, Zhang ZH, Li JB, Li LX (2022). Maternal pre-pregnancy obesity combined with abnormal glucose metabolism further increases adverse pregnancy outcomes in chinese pregnant women. Frontiers in Endocrinology.

[ref-20] Lipworth H, Melamed N, Berger H, Geary M, McDonald SD, Murray-Davis B, Murphy KE, Redelmeier DA, Yoon EW, Barrett JFR, Ram M, Diabetes, Obesity, and Hypertension In Pregnancy Research Network Investigator (2021). Maternal weight gain and pregnancy outcomes in twin gestations. American Journal of Obstetrics and Gynecology.

[ref-21] Liu K, Chen Y, Tong J, Yin A, Wu L, Niu J (2022). Association of maternal obesity with preterm birth phenotype and mediation effects of gestational diabetes mellitus and preeclampsia: a prospective cohort study. BMC Pregnancy and Childbirth.

[ref-22] Marshall NE, Biel FM, Boone-Heinonen J, Dukhovny D, Caughey AB, Snowden JM (2019). The Association between maternal height, body mass index, and perinatal outcomes. American Journal of Perinatology.

[ref-23] Meghelli L, Vambergue A, Drumez E, Deruelle P (2020). Complications of pregnancy in morbidly obese patients: what is the impact of gestational diabetes mellitus?. Journal of Gynecology Obstetrics and Human Reproduction.

[ref-24] Melchor I, Burgos J, Del Campo A, Aiartzaguena A, Gutiérrez J, Melchor JC (2019). Effect of maternal obesity on pregnancy outcomes in women delivering singleton babies: a historical cohort study. Journal of Perinatal Medicine.

[ref-25] Moronge D, Ayulo V, Elgazzaz M, Mellott E, Ogbi S, Faulkner JL (2024). Both endothelial mineralocorticoid receptor expression and hyperleptinemia are required for clinical characteristics of placental ischemia in mice. American Journal of Physiology-Heart and Circulatory Physiology.

[ref-26] Mustafa HJ, Seif K, Javinani A, Aghajani F, Orlinsky R, Alvarez MV, Ryan A, Crimmins S (2022). Gestational weight gain below instead of within the guidelines per class of maternal obesity: a systematic review and meta-analysis of obstetrical and neonatal outcomes. American Journal of Obstetrics & Gynecology MFM.

[ref-27] Nagler L, Eißmann C, Wasenitz M, Bahlmann F, Al Naimi A (2024). The association between maternal obesity and fetomaternal outcomes in twin pregnancies. PLOS ONE.

[ref-28] Ogunwole SM, Zera CA, Stanford FC (2021). Obesity management in women of reproductive age. Journal of the American Medical Association.

[ref-29] Otero-Naveiro A, Gómez-Fernández C, Álvarez-Fernández R, Pérez-López M, Paz-Fernández E (2021). Maternal and fetal outcomes during pregnancy and puerperium in obese and overweight pregnant women. A cohort study. Archives of Gynecology and Obstetrics.

[ref-30] Ozmen A, Nwabuobi C, Tang Z, Guo X, Larsen K, Guller S, Blas J, Moore M, Kayisli UA, Lockwood CJ, Guzeloglu-Kayisli O (2023). Leptin-mediated induction of IL-6 expression in Hofbauer cells contributes to preeclampsia pathogenesis. International Journal of Molecular Sciences.

[ref-31] Pratt A, Howat P, Hui L (2020). Maternal and perinatal outcomes for women with body mass index ≥50 kg/m2 in a non-tertiary hospital setting. Australian and New Zealand Journal of Obstetrics and Gynaecology.

[ref-32] Rasmussen KM, Yaktine AL (2009). Institute of Medicine (US) and National Research Council (US) Committee to reexamine IOM pregnancy weight guidelines. Weight gain during pregnancy: reexamining the guidelines.

[ref-33] Rumer KK, Sehgal S, Kramer A, Bogart KP, Winn VD (2024). The effects of leptin on human cytotrophoblast invasion are gestational age and dose-dependent. Frontiers in Endocrinology.

[ref-34] Sharma G, Blumenthal RS, Poppas A (2021). Is maternal obesity the achilles’ heel of sustainable efforts to reduce adverse pregnancy outcomes?. Journal of the American College of Cardiology.

[ref-35] Siddiqui A, Deneux-Tharaux C, Luton D, Schmitz T, Mandelbrot L, Estellat C, Howell EA, Khoshnood B, Bertille N, Azria E (2020). Maternal obesity and severe pre-eclampsia among immigrant women: a mediation analysis. Scientific Reports.

[ref-36] Stupak A, Kwaśniewski W, Goździcka-Józefiak A, Kwaśniewska A (2021). The influence of maternal obesity on cell-free fetal DNA and blood pressure regulation in pregnancies with hypertensive disorders. Medicina.

[ref-37] Sun M, Luo M, Wang T, Wei J, Zhang S, Shu J, Zhong T, Liu Y, Chen Q, Zhu P, Qin J (2023). Effect of the interaction between advanced maternal age and pre-pregnancy BMI on pre-eclampsia and GDM in Central China. BMJ Open Diabetes Research & Care.

[ref-38] Tabacu MC, Istrate-Ofiţeru AM, Manolea MM, Dijmărescu AL, Rotaru LT, Boldeanu MV, Şerbănescu MS, Tudor A, Novac MB (2022). Maternal obesity and placental pathology in correlation with adverse pregnancy outcome. Romanian Journal of Morphology and Embryology.

[ref-39] Tanner LD, Brock And C, Chauhan SP (2022). Severity of fetal growth restriction stratified according to maternal obesity. Journal of Maternal-Fetal and Neonatal Medicine.

[ref-40] Vince K, Brkić M, Poljičanin T, Matijević R (2021). Prevalence and impact of pre-pregnancy body mass index on pregnancy outcome: a cross-sectional study in Croatia. Journal of Obstetrics and Gynaecology.

[ref-41] Vonck S, Lanssens D, Staelens AS, Tomsin K, Oben J, Bruckers L, Gyselaers W (2019). Obesity in pregnancy causes a volume overload in third trimester. European Journal of Clinical Investigation.

[ref-42] Wahabi H, Elmorshedy H, Amer YS, Saeed E, Razak A, Hamama IA, Hadid A, Ahmed S, Aleban SA, Aldawish RA, Alyahiwi LS, Alnafisah HA, AlSubki RE, Albahli NK, Almutairi AA, Alsanad LF, Fayed A (2024). Neonatal birthweight spectrum: maternal risk factors and pregnancy outcomes in Saudi Arabia. Medicina.

[ref-43] Witteveen AB, Henrichs J, Bellers M, Van Oenen E, Verhoeven CJ, Vrijkotte TGM (2022). Mediating role of C-reactive protein in associations between pre-pregnancy BMI and adverse maternal and neonatal outcomes: the ABCD-study cohort. Journal of Maternal-Fetal and Neonatal Medicine.

[ref-44] Xiang C, Sui L, Ding X, Cao M, Li G, Du Z (2024). Maternal adiposity measures and hypertensive disorders of pregnancy: a meta-analysis. BMC Pregnancy and Childbirth.

